# Mississippi Valley Association of Dental Surgeons

**Published:** 1885-04

**Authors:** E. G. Betty


### THE DENTAL REGISTER.

VOL. XXXIX.] APRIL, 1885. [No. 4.

### PAPERS, DISCUSSIONS AND PROCEEDINGS

### Of the Forty-First Annual Meeting of the Mississippi Valley Association of Dental Surgeons.

Held at Cincinnati, O., March 4th, 5th, and 6th, 1885.

REPORTED BY E. G. BETTY, D.D.S.

The forty-first annual meeting of this time honored association took place in the lecture room of the Ohio Dental College, according to custom.

The session was opened at 10:30 A. M. March 4th, with President Van Antwerp in the chair, Dr. Leslie leading in prayer. The minutes of last year's meeting were read by Secretary Rose, and no objection being offered, stood approved. Dr. E. G. Betty, Chairman of the Executive Committee, read a written report in which it was stated that three hundred programmes had been printed, one half of which were turned over to Corresponding Secretary Berry, who mailed a copy to every member whose address he could obtain; a copy was sent to all the leading journals, and in addition, nearly a hundred letters had been written to as many members, urging them to be present, and to prepare a paper to be read at the meeting. In answer to these, some ten or a dozen papers were promised. The programme embraced the following topics for discussion:

I. Progress of Dental Education.

II. Recent Theories Concerning Caries of the Teeth.

III. New Remedies for the Treatment of Oral and Dental Diseases.

IV. Improvements in Prosthetic Dentistry.

V. New Methods and Materials for Filling Teeth.

VI. Incidents of Office Practice.

On motion of Dr. Sillito the report was accepted and adopted. Dr. Taft, Chairman of the Publication Committee, reported that the proceedings and discussions of the fortieth meeting had been published in the "REGISTER" as usual. Dr. Cassidy proposed Dr. W. D. Phillips, of Cincinnati, for membership, who, on motion of Dr. Betty, was elected by the Secretary's ballot. Dr. Berry moved that the time of of the sessions be fixed at from 9 a. m. to 12 m., and from 2 p. m. to 5 p. m., and from 7½ p. m. till adjournment. The night session was stricken out, and the motion passed. The first subject on the programme was now announced by the President, "Progress of Dental Education" and papers called for. Dr. Arnold responded by reading the following:

The first subject on the programme ought to be of sufficient import to interest every practitioner and develop some suggestions based upon observations and facts, to stimulate a healthy sentiment for the perpetuity of everything that will strengthen and add to the usefulness of our calling.

From the earliest history of dentistry, men, eminent for their ability and, strong in their solicitude for the future of our profession, have taken an active part on this question, and much wholesome and sound doctrine has emanated from this source; and if we were only more ready to refer to the sentiments, as expressed by these earnest workers for the good of our cause, and as set forth in many publications of the past, we would conclude there was very little more to say—even to-day. But as there is a demand for something new, so called, I will endeavor to commit a few thoughts to paper, at the risk even, of reiterating much that is old—in sentiment at least. The subject would naturally suggest the query.

Have we been progressive?

Yes! We have progressed continously from the day the turn key was the universal remedy for all dental troubles until today—the most delicately made and artistically finished forceps lie comparatively idle in our cabinets and teeth are saved rather than lost. From the day teeth were filled with lead and fusible alloys; when exposed pulps were never saved; until to-day we manipulate gold at will, and living pulps are rarely destroyed. From the time the origin and cause of dental caries were merely matter of conjecture—until to-day we have an intelligent classification of the destructive elements that enter into the various forms of dental decay, and, we are in a measure able to arrest their progress. From the time dental colleges were unknown until now, institutions are scattered throughout the land, with their well selected corps of instructors, affording abundant opportunity and the best facilities for instruction to all who may apply. Then again, our numerous societies and periodicals, neither of which is half a century old—furnish a source for information that is simply inestimable.

With all this fresh in our minds, all must admit that we have been progressive. But, notwithstanding this is true, I believe we are still too circumscribed, and that our field of usefulness must be enlarged. For fear of making what was intended to be a short paper, a long and tedious one, I'll be brief and to the point.

I have frequently been struck with the manifest indifference and limited knowledge among members of our calling, concerning the sciences of Therapeutics and Materia Medica, and the art of prescribing. If we are to maintain our claim for professional independence, or as specialists of a learned profession, more progress must be made in this direction. No one will deny the importance and necessity for medication, many dental lesions demand. The first symptoms of dentition in the infant often call for the most urgent treatment; and the eruption of the dens sapientiæ is not wholly free from complications demanding judicious medical aid.

Are we not too often guilty of criminal negligence by a limited application of our services; ignoring remote complications, and utterly disregarding general medical principles? Are we not too willing the general physician have charge of everything beyond the mere manipulations that are almost exclusively mechanical, and quite limited as to territory involved? If we are not all full fledged medical men, we, at least, must be dental physicians and surgeons; and in this way only, can we hope to elevate our calling to a still higher plane, and moreover, extend the usefulness of our profession.

We don't want to be general practitioners of medicine, in the fullest sense of the term; if we did, many of us would probably abandon dentistry; but there *is* a happy medium between extremes, and, I'll assure you, a consultation between medical and dental practitioners often develops a most successful desideratum with the most beneficial results to all concerned. The coming dentist must either enlarge his field of usefulness, or suffer the odium of ostracism to any claim as a member of a learned profession.

Recently while reading a novel by a popular author, I was impressed by the following remarks, which are quite apropos to the subject under consideration.

"There is a class of commonplace intellects who regard knowledge of all kinds in the light of a ladder—one ladder for each science—and the rungs of the ladders are the successive facts mastered by an effort, and remembered in the order they have been passed.

"These persons think it is possible to attain to high eminence on one particular ladder--that is, in one particular science, without having been up any of the other ladders--that is, without a knowledge of other brances of science.

"This is the mind of the plodder, the patient man who climbs, step by step, in his own unvarying round of thought; not seeing that it is but the wheel of the treadmill over which he is laboring, and that though every step may pass, and repass, beneath his toiling feet, he can never obtain a bird's-eye view of what he is doing, because his eyes are continually fixed on the steps in front."

The application of the above can readily be appreciated, when we look about us and see the large number of dentists who are content to know nothing more of science and art than is absolutely necessary to perform commonplace operations limited to filling and making artificial teeth."

*Dr. Berry*—I also have a paper on this subject, though it is as yet not quite complete, and I would like to have a little time in which to finish it while the members are discussing Dr. Arnold's paper. I would like to say right here, however, that I am particularly impressed with Dr. Arnold's statement of the want of proper diagnosis of the diseases brought before us, as it is a most important element in the intelligent treatment of them. Many things are often said by dentists with nothing of a scientific nature upon which to base their statements. Upon a correct diagnosis depends the success of our treatment.

*Dr. Sage*—It seems to me that for a long time the dental profession has not availed itself of the opportunities offered in its written literature, that would elevate it to the level of the medical profession. The dentist should have a knowledge of many diseases of the mouth and surrounding parts, even though he might not be called upon to treat them. The physician entertains a higher regard for the dentist who exibits a reasonable knowledge of a disease, even if he takes it to the physician for consultation and treatment. It is these instances that gain for dentistry the respect of the medical fraternity. How many dentists recognize the various tumors and surgical diseases of the jaws, and are competent to treat the same?

The dentist needs to understand these things, and though he may not be a skillful medical man, yet he should have a general knowledge of medicine that can only be obtained by a liberal course of reading and study in medical science. In Germany, France, and England, the scientist is not dependent upon his specific labors for a living. In this country things are differently constituted, and we are therefore obliged to restrict our outside researches into science.

*Dr. Arnold*—The object of my paper was to stimulate an effort to get up an interest generally in science that has a bearing upon dentistry, as it is in this direction that we must look for progress in dental education. If we come across such cases as Dr. Sage mentioned, it is not our province to treat them, but we may recognize them, and refer them to the surgeon or physician, and yet be acknowledged by the medical profession as intelligent practitioners. The oculist is a specialist, and he recognizes the remote causes of diseases just as the dentist does, or ought to do.

*Dr. J. Taft*—I guess Dr. Berry has his paper finished by this time, and I think it would be better for him to read it, as we may if discussion proceeds any further, say things he wants to say, and it is not necessary to go over the same ground two or three times.

Dr. Berry then read his paper as follows:

*Mr. President and Members of the Mississsippi Valley Association of Dental Surgeons:*—

The progress of dental education in recent times is highly gratifying. Although for centuries there have been members of the dental profession distinguished for their learning and skill, still within the memory of people now living the great majority of those practicing dentistry were men of little scientific attainments. No liberal course of study, nor any standard of professional knowledge, were required to enter the profession; and no matter how ignorant any one might be, if he had the assurance to announce himself a dentist, he was accepted as such by the community. There was in the world no dental school, no dental society, no dental journal, no dental depot, and no legal enactment regulating the practice of dentistry.

In the early days dentists were isolated from each other. There was little social or professional intercoure between them. Each kept his secrets; or imparted the knowledge of any improvement he made for a consideration.

In those times if the dental student resolved to obtain a professional education his path was beset with difficulties. For his tuition in anatomy, histology, physiology, patholgy, therapeutics, and chemistry, he relied on the medical schools; where the teaching had no reference to the practice of dentistry. To acquire professional skill he had recourse to a private preceptor, who, if he had the ability to properly prepare him for practice, could usually ill afford the time necessary for the purpose. Nor was it generally supposed that much time or study were necessary to learn all a dentist needed to know.

To reduce the chaotic state of the dental profession to order, and elevate it to the desired standard, was a difficult task, requiring the united energy of educational influence, associated effort, and the power of the press.

The colleges, associations, and journals, scattered all over our land, and existing also to some extent in the old world, for promoting the interests of the dental profession, are, like the common school, of American origin. Their founders were men of sound judgment. They laid their foundations deep. They builded well. The results of their labors are remarkable. After a half century of effort to elevate the dental profession of our country, it is abreast of the professions of law, medicine, and theology.

The agencies so successful heretofore are to be relied on for further improvement.

Our score of dental colleges, affording facilities vastly increased above any other means of obtaining a dental education, have proved a great boon to the profession. Let them all imitate the worthy example of a few and demand a good English education as a *sine qua non* for matriculating students. Let them receive only those of fair mental ability, and good moral character, affording ground to expect them to become proficient and honorable members of the profession. Let them extend their terms of about five months to a much longer period. Deducting one week for their classes to get well at their studies, two weeks for time spent or lost at Christmas holidays, and the last week of the term for examinations and preparation for commencement, and there remain about eighteen weeks of each session for study. Attendance on two courses of lectures being required for graduation there are about thirty-six weeks devoted to college studies. It is not to be expected that any considerable proportion of the students will occupy much time in scientific researches during the vacations. That thirty-six weeks at college is too short a period for the dental student to become so well advanced in attainments as is desirable before engaging in practice, is clearly evident to any one at all informed on the subject. While no diploma should be conferred unless an examination shows the candidate to be well prepared, it would be eminently proper for the colleges to adopt a rule to graduate those whose acquirements warrant it after attending one session.

The numerous dental societies promote fraternal feeling among their members, and aid largely in the diffusion of knowledge, by their essays, discussions, and clinics. Care should be had that the code of ethics is rigidly enforced, and the time of their sessions fully utilized by closely following their programmes, and avoiding waste of time on unimportant matters.

The score of dental journals published in our country, comparing favorably with those on any other branch of science, contribute largely to the information of their readers, and stimulate them to attempt higher attainments. Let increased care be had in editing the journals. Let their writers generally furnish articles more carefully written and less verbose.

When there were no dental depots instrument makers were few and far between. The only dental appliances kept for sale were the key and forceps, poorly adapted to hold any tooth. A dentist who passed away a few months ago said, that when about to commence practice, he made the journey from the northern part of Ohio to this city to procure instruments. Arranging with a cutler, who is still making dental instruments here, he waited two weeks until his goods could be manufactured. The dental depots greatly benefit the profession, supplying every thing needed at once, and drawing the attention of their patrons to improved appliances and their uses.

Legislative enactments regulating the practice of dentistry have given an impetus to dental education inducing a larger number of students to avail themselves of the advantages of dental colleges. Some of these laws should be made more stringent, and strictly enforced."

A recess of ten minutes was taken to enable the Treasurer to collect dues, at the end of which time Dr. Porre read parts of a paper, giving illustrations in the diagnoses of diseases caused by dental lesions, showing the necessity for progress in this direction amongst the profession generally, and of which the following is a synopsis:

"A great many people are suffering constitutional symptoms arising from dental lesions, and it is our business to study them in order to treat them. There is no lack of ability in the dental profession, but individuals must be stirred up to take hold of the subject, and give it the time and attention it deserves. The progress of dentistry during the last quarter of a century has been phenomenal; in the colleges that educate; in the new and improved methods of operations and treatment we see a widening of the professional field. A preparatory education is undoubtedly necessary as there exists a difference between those who hold the degree of D.D.S., and those who hold that of M.D., principally because we are educated in a specialty.

We have emerged from the age of the "turnkey" and its correlatives, and the medical profession now looks to us, and depends upon us for assistance in all cases involving our department.

When the mouth is the seat of disease, and there are poisonous secretions, the latter by being swallowed, disorder the stomach and through it, the whole system. Scurvy causes a loosening of the teeth and gives rise to complications, that may have the gravest effects upon the system.

I may mention as an illustration of my point, a case of disease of the antrum. The patient is a man of good family history, his neck and face were puffed out, showing how extensive the local effects of the trouble were. The constitutional symptoms were those of pyæmia. Dr. Dawson, who had sent the patient to me, opened into the autrum from the buccal side, and discharged a teacupful of senious pus; the stench was simply terrible. The root of a bicuspid tooth was discovered, and this was the exciting cause of the whole trouble. It was removed and the antrum washed out with injections of carbolic acid, oil of eucalyptus, and chloride of zinc. The mental purturbations disappeared, and sound health was rapidly restored by tonics and a nutritious diet. Such cases as these impress upon our minds the necessity for extending our observations and studies, and teach us that pyæmia troubles more often arise from diseases pertaining to the mouth than either physicians or dentists are aware."

Twelve o'clock having arrived the association adjourned till 2 p. m.

FIRST DAY AFTERNOON SESSION.

Shortly after 2 o'clock, President Van Antwerp called the meeting to order, and Dr. Porre resumed his paper, giving other cases, and concluded by saying that "the time has come when we should exchange courtesies with the medical profession and its various specialists. It is necessary that dentists contribute more to the medical journals than they do, thereby enchancing the benefits to be derived from dentistry as intelligently practiced."

*Dr. Berry*—It seems to me that Dr. Porre is digressing, let us adhere to the programme, and have what other papers there may be upon the subject.

*Dr. J. Taft*—The subject of professional or dental education is one upon which we all have opinions, especially if it is discussed much. There is also quite a diversity of opinion; some are content to have it circumscribed; others to have it reach out and have it embrace other subjects, while others would have it embrace the whole range of science and literature. I apprehend that the day is not far distant when opinion upon dental education will be uniform. What has been the progress made in the past? As it is the custom in other branches of scientific investigation to take a retrospective view, so it ought to be in our department, as it is often necessary to do this in order to avoid difficulties in the future. In the past there existed much difference of opinion, but among the real workers in the profession, opinion was more harmonious; such men as Harris, Hayden, the Parmleys, and others, were practically a unit in their idea as to the progress of the profession, and were also agreed as to what should be done to advance education. Melancthon Rogers, Jas. Taylor, and others in the West, were men of this kind. If these men were alive now, they would still be in the advance as they were then, and we have few who would be more radical in their opinion as to the status of the profession. The Baltimore and Ohio Dental Colleges were the direct outcome of the opinions and energies of these men; it was also they who organized the associations. The American Dental Association has taken a high ground in this direction from the first, and has since maintained it more or less vigorously. A good preliminary education is a necessity to him who would be a candidate for the thorough education in and practice of dentistry. Ever since 1840 or '45, there has been a rapid and steady increase in the numbers of those engaging in the practice of our profession. A change is now taking place, owing to the influence of the journals, and the associations are making the profession take a higher position, both in science and the estimation of the public. The time was when the public did not look upon a dentist as any better than the mender of clocks and pans. There has been much discussion as to what constitutes a dental education, and much diversity of opinion is entertained upon the subject. The successful dentist, the reputable dentist, is the one who possesses good general culture and attainments. The ignoramus does not now enjoy an enviable reputation. It is absolutely impossible for any man to travel all over the world of human knowledge and equally so of science alone. Aside from this extreme view, general knowledge will facilitate the practitioner in his specialty. It is profitable to gain as much information as possible of all subjects in any way bearing upon any one speciality, therefore, let each according to his ability, attain as much as he can, and make it tributary to the work he has in hand. He who has general knowledge, has a better fund to draw upon ; his intellectual training is much better, and he is intellectually more vigorous and better able to practice any special branch of medical science. For instance in the study of Anatomy, he who has no knowledge of Greek and Latin is at a disadvantage in the comprehension of the nomenclature of the science, while he who has, understands far better, and sees more than appears upon the surface. The more a man has, the more power he will have ; his influence upon those about him will be the greater in proportion. The man with strength is the one that tells ; the one who has the most money is the most powerful among those with whom he is thrown in contact. He of the greatest physical strength (and you Mr. President, possess a good deal of that) towers above those beside him ; so it is with him who possesses the greatest knowledge and mental training. When talking upon this subject we are apt to forget that men differ in capacity ; some are filled with a pint, others will hold a gallon. A man asks "what shall I study?" It is best for that man to do that for which he is best fitted by nature, and in which he can accomplish the most good. The man who has perfected himself in artificial work is just as worthy of honor as he who devotes himself to surgery and the treatment of disease. The one who works not in the line for which he is best adapted wears himself out in the struggle.

*Dr. A. O. Rawls*—I did not come prepared to speak upon this subject, though I have my views upon it. I have seen the progress made as shown by the journals, associations, etc., and am satisfied with it as compared with that made in the other professions. I am really proud to be a member of the dental profession just on this account, and simply because we have gone so far and rapidly ahead of our original standard. The progress has not only been rapid and systematic, but it has gone ahead of all other professions and stands at the head upon the topics pertaining to it, and the science, literature, and art in which it has displayed such a phenomenal advance. In the science of biology for instance, who is ahead to-day? Is it not the dentist? Where did we get our splints and plastic operations? from medicine? No!

We are apt when noting the standard of dental education to consider the standard of the colleges as all sufficient for the position we occupy, but it is not so. Dr. Taft represents one of the very best colleges in the world, and he sends out students, who, in many places could not live if they conducted themselves strictly as they were educated. This is because the public is not by any means sufficiently educated to appreciate the higher aims of the profession.

*Dr. H. J. McKellops*—I neither expected nor intended to say anything upon this subject when I came to the meeting of this, the *oldest* dental society in the world, and of which every one should be proud to have his name on its record. I wish that God had given me the power and the language to express the thoughts I entertain upon this subject. We must be a profession by *ourselves* or not be *anything*. I entertain a great interest for dentistry and dental education. Does mere graduation make a man a doctor? Does mere graduation in a dental college fit a man for diagnosing diseases of the mouth? I wish every man would take up and read the life of John Hunter; *he* was a man who took *no* one's word but studied for himself, and discovered that which made him a dentist. It is the associations that I have traveled about the country to visit that have built me up.

What was Patrick forty years ago? he was a diamond setter; what is he to-day? one of our most prominent and honorable men in the profession. See what a man Buckle was! Read his History of Civilization in England, and note the great mine of wealth of knowledge he possessed; every page teems with it, the breadth of his researches into the history of man was almost without limit; he was a man of great brain, and he used it. *That's* the kind of men we want. If we are going to be a profession let us *be* one, and not tackle on to *anything*. We have competent men and professors, and we must educate ourselves. I *love* dentistry, if I did not, I too would be a stay-at-home, would visit no associations; wouldn't I rather remain at home, mix up a little a-mal-gam in a cup, and think it was the little Jesus Christ that revolutionized the world? The revolution we want is in the *clinic room;* the man we want is he who goes into the clinic and *demonstrates, not* him who writes unintelligible symbols on the black-board.

Oh, I wish I was one of those—Methodist fellows, one of those exhorters, who can tell the thoughts within them, and can carry conviction to the minds and hearts of their hearers. Gentlemen, I thank you for your kind attention. [Applause.]

*Dr. Sage*—I am not opposed to a liberal education for the dentist, but I am opposed to a smattering of many things. I believe in concentrating energy upon one thing and in one line.

*Dr. McKellops*—I wish to God that I was one of the distinguished men of the dental profession. Dentists did not have to go across the water to bring dentistry here. No sir; we *teach* those men over there, and they look to this country with breathless anxiety for every flash of the electric light of genius that sparkles from our distinguished men. What do their journals there amount to? They are an almost exact reproduction of ours. If we sit down and are content to earn a living only, we shall never become enlightened; for that, we must read, study, search into nature and discover her living truths. The men who do that are the ones who progress and reflect their light upon us all, and they are the kind we most want.

*Dr. Berry*--I fully agree with our distinguished visitor from St. Louis; I ought not to say visitor either, for he is one of the older but not the earliest, members of this association. When I read Buckle's "History of Civilization in England," I too was amazed at the vast research it evinced, and in this connection I would say to the young men, read and study every book that has any bearing upon your special branch.

*Dr. J. Taft*--No one has advocated the study of all science, but no one can have any objection to a reasonable preliminary education. Some reference has been made to the colleges as being responsible for the status of the profession. Bear in mind that the colleges are restricted in the results they can obtain; up to a certain point they lack support. They have gone on as fast and as far as they can be supported and sustained, they are inclined to make education as advanced as they can in proportion as they are sustained by the profession. At Saratoga Springs last August, the Association of College Faculties* decided that all students should be examined in the ordinary branches of an English education, and that a graded course of instruction be adopted, with an intermediate examination. I refer to this action of the colleges to show that definite progress is being made; that the colleges are conferring and working together, and that this will be productive of great good. When the students come prepared with a good English education *then* the profession will have accomplished a great advance; it is coming, the public requires it, the other professions require it, and it *will* come, Mr. Chairman.

*Dr. McKellops*—I approve of all my friend Prof. Taft has said, but in my estimation it does not strike the keynote. It is impossible for the colleges to give a fine demonstrator a sufficient fee in order that he might live upon it, and consequently devote his entire time to demonstrations. *This* is where the colleges miss

---

*See the report of the proceedings of this association in the September number of the REGISTER for 1884, p. 436. E. G. B.

it. If men are not thoroughly drilled, they cannot be competent. If a man is drilled by a competent and capable demonstrator, when he goes out of college he will have acquired that which is most necessary. The *clinic* is where the young men should be taught and *thoroughly* taught.

*Dr. Rose*--I wish to say a word for the young men. They are cried down by everyone, and said to know nothing, whereas the contrary is the case, for they are tolerably well posted, and can do good dentistry.

*Dr. G. W. Smith*--The trouble is that when we leave college we settle down into carelessness, and do not keep on with our studies. The office is a good training place for students, and dentists could help the faculties by taking students.

*Dr. Porre*--A fair literary education is necessary, and if any man without it enters the profession, it is the fault of his preceptor. There is a future before us in the profession, and in the near future a great change for the better. We have reached that point when it behooves us to make better history.

*Dr. Leslie*--In Europe there is a total lack of the desire so common here, to be recognized as practitioners of a branch of medicine.

*Dr. G. W. Keely*--I see that my friend Dr. McKellops sympathiscs with the demonstrators. I doubt if there is a dental college in the United States where the teachers are able to live comfortably upon the salaries paid to them. A great point in the line of progress in dental education would be to abolish the colleges as they now exist, and in their stead found four great schools, one each in the North, South, West, and on the Pacific Slope. [Applause.] We could then concentrate all our energies upon them, endow them richly, and thus be able to employ competent teachers who would be well paid, and could then devote their whole time to educating and preparing students for the thorough and scientific practice of their profession. Laws should be enacted *requiring* students to prepare themselves in these colleges; then we would have a great and powerful means of elevating the profession to an exalted standard."

The Treasurer, Dr. Frank Hunter, reported that the books were missing, and that he could not make a report from them, but had papers and vouchers, and the funds. Adjourned till 9 o'clock A. M., Thursday.

SECOND DAY, MORNING SESSION.

The association was called to order by First Vice President Sage at 9:30; the minutes of the previous day were read and adopted. Dr. Cassidy proposed Dr. J. E. Morton, of Brookville, Ind., for membership; the gentleman was elected by Secretary's ballot. President Van Antwerp now arrived, and assumed the duties of the chair. The first subject was passed, and the second "Recent Theories Concerning Caries of the Teeth," announced. As all the members had not yet arrived the subject was temporarily laid over to allow Dr. Kempton to read this paper:

Several years ago, a pharmaceutical student, viewing the rapid approach of examination day with apprehension, and lamenting the numerous difficult studies he was required to master, said to a student of mine, "I am sorry now that I took up pharmacy instead of dentistry, for all a dentist has to learn is about the teeth and gums."

This may seem absurd but his opinion of the attainments of the profession is shared by a goodly number of the laity, humiliating though it be, and yet when we consider the calibre and qualifications of some of our professional brethren we cannot blame them much. The teeth and the gums may seem a small matter, but when we consider the number of individuals whose daily occupation is the care of these organs, it is evident that they are important parts of the human economy. That this regard for them is not of recent origin is shown by the sayings of several old writers, among whom is Cervantes who says: "I had rather they had torn off an arm, provided it were not the sword arm; for thou must know, Sancho, that a mouth without teeth is like a mill without a stone, and that a diamond is not so precious as a tooth." But that, however, was before the advent of those Good Samaritans who make a brand new set of teeth for $5, no charge for extracting. Now gentlemen when we consider the amount of literature that has been, and is being published on this subject, it would seem that about all that was possible to say had been said, and anything further would in all likelihood be a rehash of known facts. So you need not be surprised if what I may say is not new to you.

Amongst all the heated discussions in regard to stopping the ravages of dental caries, the best material to do it with, and the best method of manipulating said material, we occasionally find some one who, "like the voice of one crying in the wilderness," calls our attention to the poor texture of many of the teeth we meet with, and questions whether it is possible to improve it. Others who have taken up this thought and investigated it, tell us the trouble is a deficiency of phosphates in the enamel and dentine, probably due to a lack of these principles in the food, and as beans, oatmeal, cracked wheat, rice, and graham flour are rich in these salts, consequently all that is necessary to do in order to build up a perfect set of teeth is that the mother during pregnancy and lactation, and the child after being weaned, should partake of a diet composed largely of beans, oatmeal, cracked wheat, rice, or graham flour, a la Dio Lewis.

We have all seen children whose deciduous molars have been swept away by caries before the eruption of the first permanent molars, whose incisors were soft and carious, whose muscles were flabby or mushy, and perhaps we have advised a diet of beans, oatmeal, cracked wheat, rice and graham flour, and have been told that such a diet had been attempted but the children rebelled against it. Perhaps we have tried such a diet on ourselves, only to give it up in a short time in disgust to return to the flesh pots of Egypt, concluding that the only reason the "Heathen Chinee" flourishes on such a diet is because he *is* a heathen.

If, however, we investigated the method of preparing these cereals for the table, we are struck at once with the astonishing stupidity of the average cook, and realize the truth of the saying: "The good Lord gave us the food but the devil gave us the cooks." We find that the rice, oatmeal or cracked wheat, are put in a stew-pan with an excess of water, placed on a hot stove and stirred every few minutes to keep from sticking to the vessel. The result is a pasty mass that is apt to be scorched, and sure to be unpalatable. If instead of this method the cereal was allowed to soak in a sufficient quantity of water till it had absorbed as much as it could, was then put in a tin vessel with a little water, and such seasoning as was deemed proper, placed in a water bath, and cooked a certain number of minutes, the result would be that the children would not only not rebel against it, but demand such a diet, for it will be found that the natural flavor has been retained, and the grains are whole instead of being in that disgustingly pasty condition usually seen. But gentlemen, we sometimes meet with persons in whose mouth, judging from their diet, we should expect to see teeth of adamant, but find instead the poorest kind. This compels us to seek some other cause than bad food, and leads us to the conclusion that defective assimilation should bear part of the blame.

What is assimilation? It may be defined as that process which turns grass into hair when eaten by cattle, and into wool when eaten by sheep. It is to the body what the workmen are to the building, and it matters not how much of the finest Italian marble, how much thoroughly seasoned ebony, rosewood, mahogany or other fine woods, how much iron, stone, lime, and other things necessary to the contruction of a building are carted up and dumped down on a building site if the workmen are either on a strike, or are unskilled, for the building will surely be unsightly and liable to perish when the storms beat and the winds blow.

Assimilation, the function that is so important to life, is favorably influenced by several things, the most important of which is sunlight. We of this enlightened nineteenth century, who think that more progress has been made in it than in all the time preceding, are wont to look down with pity and contempt on the ancients, who bowed down in reverence and worshipped the glorious orb of day. But it is evident that they recognized in the sun the source of all good, perceiving that only bats, lizards, and such kindred vermin flourished in darkness. We, on the other hand in spite of our boasted intelligence, build our houses with little reference to light, and the windows too small by half are obscured by stained glass, lace curtains, or still worse by those rag carpet resembling Portieres ; the rays of the sun are regarded with prejudice, as they cause their carpets to fade. People who would never think of trying to cultivate plants under such unfavorable circumstances, keep their children in such places, and then wonder that they are anæmic, pale and sickly.

Another thing influencing assimilation is pure air, air that is not mixed with the emanations from filthy gutters, fermenting garbage piles, sewer gas, or the products of combustion that escape from the illy fitted joints in stoves or stovepipes, or the exhalations from the lungs in crowded compartments, in other words good hygienic surroundings. Still another influence is exercise, which is illustrated by the oft cited example of the blacksmith's arm. Exercise that calls into play a variety of muscles, creates a demand for nutritious food, which after being digested, and while circulating through the capillaries, is seized on with avidity by the various tissues, and incorporated in their substance.

In order to be of benefit the form of exercise should be such as to convey pleasure to those exercising, for unless they are interested in it they will regard it as a task, and not indulge in it. It is, however, when these three conditions, viz: sunlight, fresh air, and exercise are combined, that we get the greatest good from either, as one can readily see by passing along through our markets and observing the translucency and ruddiness of the complexions of the market men and women, while on the other hand the sallow, worn, and jaded appearance of those who work in factories indicate a lack of pure air and sunlight.

The conditions referred to above favor the production of good tooth structure in as much as they favor improvement in all the tissues, but there are certain local conditions to be taken into consideration, the principal of which is use of the teeth. It is a well known fact that use of an organ promotes growth, whilst disuse results in atrophy, and reasoning from analogy we conclude that the teeth are amenable to the same rule, and that the mania for chewing gum that is so prevalent among children, should be encouraged instead of being tabooed.

In reviewing the unfavorable influence, we find first heredity, over which, however, we have no control, and so pass to the consideration of the next which is the drain of nerve force, due to a premature stimulation of the mental faculties, as seen in children who have been sent to school too soon, in whom the nerve force that should go to the development of the muscular and osseous systems is diverted to the cerebrum, leaving the muscles and bones to get along the best they can, the result being a developed brain mounted on an impoverished body, that is supported by pipe-stem legs. The teeth are like chalk, and the muscles almost imperceptible. When such a being enters the race of life, and competes in the struggle for existence it soon succumbs.

In summing up we come to the conclusion that in order to improve the structure of the teeth we must begin at the beginning, and endeavor to improve the general condition. The young infant should be taken out airing every day (unless the temperature is in the neighborhood of zero) in order to get the full benefit of air and sunshine, and from the time it is old enough to walk, should be allowed to play out doors as much as possible. The windows of sleeping apartments should be opened for a certain length of time every day, in order to thoroughly ventilate them, and drive out all the excreta that may be impregnating the air.

Furthermore, children should not be sent to school too soon, probably not till they are eight, and surely not till they are seven years old, then the muscular and osseous systems are so far advanced that they can keep pace with the cerebrum. Again, let them chew all the wax they want to, and give them food that will exercise their molars.

This, gentlemen, is what ought to be done, but unfortunately too many of the laity regard us as mere menders of the masticating machinery, and pay little attention to our words, and seem to think the cobbler should stick to his last. Still there are some who pay attention to us, and profit by our precepts, so we should keep on and do our part towards promulgating knowledge."

Dr. C. M. Wright now read the following paper on the "Development Hypothesis":

If the "Development Hypothesis" of scientists be true, and if it can be shown that any existing species—animal or vegetable—when placed under conditions different from its previous ones—*immediately begins to undergo certain changes of structure fitting*

*it for the new conditions*; then, it seems to me, we may be able to place dental caries in its true position in the economy of the universe. As the nervous system of man developed as he became more erect, and the facial angle enlarged in accordance with a condition where *intellect* is the predominant feature, the teeth naturally lost their value as weapons of defense against destructive enemies, and also as means of securing food for the system. We can well understand then that by almost imperceptible steps in the slow process of evolution, the conditions being changed, the structure of the teeth began to deteriorate as the nervous system developed. The dental profession has for a long time recognized dimly the relations between the nervous system and the teeth of man, and this hypothesis that we have referred to, may account more fully for the existence of the dentist too, than any other. If we accept it, is it not easy to infer that we are fighting with our frail excavator, burring engines, gold and porcelain, against the inevitable? And does not "dental caries" assume a more gigantic form that even our wildest imaginations have ever given to it? Can we hope simply with our skill and remedies to stay the mighty wave of Law and Nature that has been gathering impetus and volume for millions upon millions of years? How we pity or laugh at the child who vainly tries to hinder the rising tide of the ocean with his little dams of sand upon the beach? Yet this is what we are doing if we believe in the law of progress, when we try to stay the Juggernut Car of dental caries. Teeth of man must go. The conditions have changed. The relation between mind and teeth is tolerably clear, and as man advances in the qualities that make him great, his unnecessary appendages, unnecessary for survival in the struggle of life; must go. Would it not then be more philosophical in us to accept this fact, and define our duty clearly in our own minds. That duty would be, it seems to me, to regard the comfort and the ease of our patients, to alleviate pain, and to waste no energy in attempting the impossible, or in building flimsy air castles of hope for a future of *improved* dental organs in man."

*Dr. A. W. Harlan*—I listened to the reading of Dr. Kempton's paper with considerable interest; we cannot overestimate the necessity for out door exercise. It is difficult to overestimate the value of rules for guidance in the selection of proper food, suitable exercise and other hygienic measures, as for the lack of these the teeth suffer as well as the rest of the body. The question of assimilation is a most important one, and how we may best prepare the body for that function is one part of the exercise of our professional duties. The observance of correct principles of hygiene--no over-stimulation of the development of the intellect--enters into the formation of good dental organs more than does the administration of specific foods. I do not take that gloomy view of the decay of the teeth of the present generation that many do. The observation of the teeth of prehistoric and ancient people, and those of two or three centuries ago, does not show that ours compare unfavorably. We see many thousands more teeth than our forefathers did, and we endeavor to save the whole thirty-two, while they contented themselves with eighteen or twenty. All the pounds of gold, tons of amalgam, and thousands of ounces of cements and gutta percha, testify to our efforts in saving thousands of teeth, where formerly but one would be attempted, so great is the disproportion. It is not uncommon to see teeth that were filled thirty or forty years ago and saved; they could not have been better teeth than those of to-day, so we should continue and persevere in our efforts for the conservation of the organs of mastication. Our high aim in the future is to discover the causes of disease and take preventive measures accordingly.

*Dr. Wright*—I am sorry if my paper has created the impression that it is useless to try to save teeth for there are millions being saved every year. I referred to the great broad view of the subject from which it seems the tendency is to destruction.

*Dr. Berry*—I wish Dr. Miller was with us so that he could tell us all about the little bug that is instrumental in producing the decay we are wont to attribute to the acids.

*Dr. J. Taft*—I fail to see the availability of discussing what will or will not happen five or six million years hence and do not see the necessity of discussing this retrograde process Dr. Wright mentions. Why should the teeth suffer more from retrogression than the eyes or any other organ of the body? Our work is with to-day and to arrest decay as best we can and assist in producing the best development of the body possible. Specialists confined to one subject are very prone to overestimate their especial department. What course of development can you specify for the building up of a given organ or a part without producing a corresponding effect upon the whole organism? The architect of a building might just as well confine his attention to the windows only, instead of directing himself to the production of a complete and symmetrical structure. All parts of the system must be cared for together, otherwise we will fail in gaining our object.

*Dr. Wright*—I am sorry to hear that Prof. Taft does not care for harmony and law of nature. I thought we might get down to something positive, if we took the broad and liberal view of the whole subject of development.

*Dr. Taft*—I deny that Dr. Wright's premise is a "law of nature."

*Dr. H. A. Smith*—Dr. Berry hinted that he would like to hear something recent in regard to theories of decay. There is a more recent theory than Dr. Miller's. It is that of Dr. G. V. Black in which it appears that those cases we have called "abrasion," near the margin of the gum, are caused by the secretion of a solvent which causes the wasting away of tissue. I saw a case of an inferior cuspid; the tissue of these teeth is particularly dense and yet the wasting away occurred one-third way up the tooth against the action of gravity. What is this? Is it a ferment? Another peculiarity is that the nearer the approach to the pulp, the softer the tissue, in other words, the further you get from the enamel, the softer is the tissue. I would like to have this point explained.

*Dr. McKellops*—This question came up at a social gathering at which Dr. Black was present. I see these cases all the time. One, a lady, aet. 30 years, softening and wasting away of the molars; her teeth are well taken care of and she had good work done upon them. Since she came under my notice, she has been married and become a mother; the enamel upon her molars and bicuspids is all gone and wasted away without decay. Another case, a gentleman—all work upon his teeth failed to save them, gold failed as did amalgam; his wife, of German descent, is rosy and robust, her teeth go the same way and in treating these two cases I found that oxyphosphate is the only remedy that gave any proportion of success.

*Dr. H. A. Smith*—I wish to get this particular cuspid tooth well before the meeting as I want to learn something definite about it. Does the withdrawal of nutrition from the dentine soften it and allow it to waste away? Is it right and honest to fill such cases and promise that the tooth will be saved?

*Dr. G. W. Smith*—Dr. H. A. Smith is ahead of the subject on the programme when he refers to filling teeth.

*Chair*—As I understand it, Dr. H. A. Smith is trying to get at the very point of the subject.

*Dr. Sage*—I have seen many cases of extreme sensitiveness, but none so bad as to need extraction.

*Dr. H. A. Smith*—In the case I mentioned there are no acids and no micro-organisms so far as can be discovered.

*Dr. Sage*—Dr. Smith's case and Dr. Black's theory are both very interesting. The question of assimilation of food has a direct bearing upon the question of caries of the teeth.

*Dr. G. W. Smith*—The recent theory, promulgated lately in Germany, is to the effect that innumerable micro-organisms are found throughout the mouth, upon the teeth, and——

*Dr. Sage*—Mr. President, I protest, and call the gentleman to order, he is trying to foist the old Leptothrix Bucallis theory upon us. (Laughter.)

*Dr. G W. Smith*—No sir, I am not; what I say is true and new, and if the gentleman will read up he can inform himself a little. (Laughter.)

*Dr. Berry*—Will Dr. Smith (G. W.) be kind enough to inform us in what way and how the bugs set up this putrefactive process?

*Dr. G. W. Smith*—By——putrefaction, of course. (Laughter.)

A motion was then carried to pass the subject and the third, "New Remedies for the Treatment of Oral and Dental Diseases," was announced.

Dr. A. W. Harlan read the following paper upon the subject:

Cocaine Hydrochlorate.—I will not burden you with the Materia Medica and pharmacy of cocaine, as all of the recent numbers of medical and dental journals have teemed with papers and notices of this new local anesthetic. From an experience of several months use of cocaine I have found that the weaker aqueous solutions, two and four per cent., are nearly valueless for obtunding sensitive dentine in superficial cavities. A ten per cent. aqueous solution is effective when retained in a cavity for a period of ten minutes or longer. A ten per cent. solution in eugenol is even more valuable, and I prefer the latter for use in deep seated, sensitive cavities. When it becomes necessary to remove the pulp, a ten per cent. solution in ether is preferable, as the rapid evaporation of the ether enhances the local anesthetic value of cocaine. In all superficial operations on the soft tissues of the mouth the weaker aqueous solutions are to be preferred as they are sufficiently potent when the soft parts have been dried and the surface to be operated upon is penciled and the solution allowed to remain undiluted for a period of from two to ten minutes. A drop of the four per cent. solution injected into the pockets, in pyorrhoea alveolaris, lessens the pain which results from the removal of small deposits on the roots of teeth. Cocaine is a styptic on account of a peculiar tannate which is found in the leaf. It is possible to extract several teeth by its deep injection in the inferior maxilla at the mental foramen or at the entrance of the inferior dental canal. In such cases the inferior dental nerve and its branches appear to lose all sensibility. Reference to its dental uses is here mentioned in order to draw out a full discussion.

Eugenol.—The writer called attention at the late meeting of the American Dental Association held at Saratoga to the above mentioned drug. A further use of it has demonstrated beyond a doubt that eugenol will supercede all other medicaments for the treatment of alveolar abscesses with a fistulous outlet. I have used it in preference to carbolic acid, creosote, solutions of chloride of zinc, aromatic sulphuric acid, creosote and iodine, eucalyptus, and numerous other drugs formerly used for this purpose. As a dressing for root canals after the removal of the pulp, whether putrescent or not, no other drug is so agreeable or satisfactory to the patient. It is a potent germicide. I use it in full strength or diluted with oil of cinnamon, sassafras, gaultheria or some other agreeable essential oil. By making a creamy paste of iodoform in eugenol we have an elegant dressing for an exposed or nearly exposed pulp. The same paste may be packed into an inflamed pyorrhoea pocket with certainty of reducing the inflammation and permanently arresting the pain. Eugenol, oil of cinnamon, and crystals of carbolic acid, ʒii. of the two first mentioned, ʒi. of the latter, applied to an aching tooth, will subdue the most refractory odontalgia. Eugenol is not gummy, deposits no sediment, does not deteriorate by age, and is not expensive.

Sanitas.—The oxidized oil of turpentine contains from ten to twelve volumes of peroxide of hydrogen, which is permanently stored up for future use. I use it in the treatment of all blind abscesses and for injection into the pyorrhoea pockets when an astringent is not indicated. In the latter cases I add either eugenol, oil of cinnamon, or sassafras, one part to three of sanitas. They disguise the odor of the turpentine, but do not wholly obliterate it. As a germicide and disinfectant in dental practice it should be very generally used. It does not injure an instrument or roughen or excoriate the hands as the bichloride solutions do. It is not poisonous except in large doses; an ounce would not destroy the life of an adult.

Mr. Ringyett, in a late number of the *Medical Times and Gazette*, London, who is the discoverer of Sanitas, says:

"1. Oxygen (a non-poisonous substance) acts as a germicide to all micro-organisms classified by Pasteur as anacrobies.

2. Peroxide of hydrogen (a non-poisonous substance) has been proved by my own investigations to act as a germicide to micro-organisms that originate putrefaction, and my results were subsequently confirmed by Guttman and Froenkel. They were also confirmed by Mr. M. M. Hamlet, who observed that of all the many chemical substances he examined, peroxide of hydrogen acted most fatally to bacterial life. Further proof of the germicidal character of this substance has been given by M. Baldy, and emphatically by M. M. Paul Bert and Reynord. Sanitas in all its forms contains or yields peroxide of hydrogen.

3. Thymol can scarcely be called a poisonous substance, and it is admitted to be a powerful germicide. Sanitas contains thymol.

4. It does not follow that, because a chemical agent is non-poisonous to man, it will prove harmless to micro-organisms. There is nothing in common between the other two."

This paper has been republished in the *Dental Cosmos*, page 757, *et seq.*, for December, 1884. I agree with Mr. Ringyett as to the value of sanitas. My method of using sanitas is to let it precede the entrance of a drill into the pulp chamber when I am opening it. The rubber dam is in place; I do not allow saliva or other liquid or solid to gain entrance to the pulp canal before it is bathed in sanitas. The putrescing pulp or its remains is removed under copious dressings of the oil. Instruments are dipped into it previous to cleaning the canals. After all debris has been removed the roots are packed loosely with cotton moistened in it. The cavity is sealed with gutta percha, through which two or three minute openings are made for the escape of any gas accidentally enclosed in the canal or beyond its apex, when there is a blind abscess. The dressing is removed in four or five days, previously adjusting the rubber dam. The roots, if not already clean, are cleansed thoroughly, and they are redressed. After the expiration of a week the dressing is again changed and the roots stuffed with shreds of cotton, wet with sanitas. In another week the roots are usually ready to be filled permanently; if not, the same treatment is pursued as previously outlined. You are in no danger of inciting an acute abscess if the treatment has been carefully followed step by step. Those operators who dread to open into a pulpless tooth, with no fistulous outlet except through the canal, may by first cleaning the sac at the apex of a tooth with $H_2O_2$, proceed with sanitas and have no after evil results, if they will practice with efficient medical agents and use intelligence and judgment.

Canabis Indica.—For a very short period I have been experimenting with the tincture of canabis indica of the strength of ten ounces of the tops of the plant to alcohol one pint. So far I have succeeded in removing one pulp after a saturation of it for five minutes. I have also excavated several cavities, very sensitive, by using the tincture as above. The pain of cutting seemed to be less intense, and did not produce much discomfort to the patient. I allow it to remain in the cavity for several minutes and then proceed as usual. I only call attention to it at this time to induce others to experiment in the same direction."

*Dr. Sillito*--Does Eugenol do away with the odor of Iodoform?

*Dr. Harlan*--Almost entirely.

Dr. J. S. Cassidy then read a paper on Cocaine as follows:

So much has been printed lately in regard to cocaine and its salts, that I almost hesitate to call the attention of this association to the subject.

When Nieman, in 1860, first fully described the active principle of the leaves of eupthroxylon coca, the alkoloid did not receive much attention from the medical world, although the leaves of the plant had been, from time immemorial, the chief source of delight to the Peruvian Indians, as a mild masticatory nervine stimulant.

Biddle, in his Materia Medica, edition of 1883, mentions the discovery of the alkaloid *cocaiana*, but prefers the fluid extract, as the best preparation of coca, and indicated only in diseases requiring the checking of tissue waste, as in phthisis. Its effects on man are described as producing cerebral stimulation, lessening of the feelings of fatigue, hunger, and thirst; increased cardiac action, and elevation of temperature; lessening the excretion of urea, and finally dilatation of the pupil, which latter effect by the way, suggests the non-interference by the drug with the sympathetic nerves. So much for its internal physiological influence.

Its use as a local anaesthetic, first publicly exhibited by Dr. Koller of Vienna, a few months ago, has given it such celebrity in all branches of minor surgery, that each one of us must recognize its importance, and give it a fair trial in our own department. With this object in view, and with the hope of exciting discussion by presenting the results at this meeting, we selected a four per cent aqueous solution of cocaine chloride.

Case 1.—Unmarried female, of lymphatic temperament, aged about 25 years, presented both superior centrals with proximal cavities of white variety, dentine extremely sensitive. Made two applications of the solution and in about ten minutes, found the dentine almost devoid of sensibility.

Case 2.—Superficial anterior proximal cavity, third inferior molar, in man about 40 years old, sanguine temperament, addicted to chewing plug tobacco. He would *not* submit to the torture inflicted by the excavator; had recourse to our pet, three consecutive times, at intervals of about five minutes, with no apparent benefit whatever. Was failure in this case due to the saturation of the part with nicotine?

Case 3.—Youthful patient squirming with the pain caused by adjusting clamp; applied cocaine solution freely, and worked as heroically as before, the patient showing no evidence of suffering.

Case 4.—Female aged about 20 years, nervous temperament, first superior bicuspid presenting near the neck a carious anterior proximal surface, extending nearly half way across the labial surface. The hypersensitive condition of the part indicated the use of an obtunder. After three applications of cocaine, the patient declared the excavating did not hurt in the least.

Case 5.—A partially devitalized pulp was bathed effectually with the (in this case so called) anesthetic, and no decrease whatever in sensibility seemed to result.

Case 6.—Male, 35 years old, nervous sanguine temperament, anterior proximal cavity in upper cuspid, of common variety in color, depth, and position, and labial small cavity in neck of first lower bicuspid. The dentine in each so tender that he appeared to suffer absolute torture by my effort to excavate. Made one application of cocaine solution on cotton wool, and let it remain perhaps twenty minutes, while I repaired a filling in one of his molars. My patient was a thorough scientist and had no confidence in the efficacy of the drug; but when I again began cutting with bur and drill and excavator, his enthusiasm in its favor, knew no bounds, and I am positively sure he suffered little or no pain.

These are but a few examples of similar experiments—with successes more than less—sufficiently suggestive, and warranting the question: are the virtues of cocaine, as a dental anaesthetic dependent in any way on the temperament or idiosyncrasy of our patient; or, are its numerous failures due to some unknown local conditions that are able to resist its obtunding influence?

Dr. Betty of this city in the February No. of the *Register* says, that vascularity seems to be an essential condition, and that its effectiveness is due to its ability to cause anemia; but Dr. Arnold of Ironton in the same No. of the *Register* presents several instances of complete anaesthesia of dentine by its use.

It would seem therefore that it must act on non-vascular tissue by a direct paralyzing effect on the peripheral sensory material; or would it be more in accordance with present histological views, to attribute the loss of sensibility to a change in wave motion, either an increase or diminution of the normal vibration in the nearer portion of the mass of protoplasm? It is evident at all events, that in as much as no cauterizing effects are produced, the expectation of employing it with invariable success on sensitive dentine, or even vascular tissue, must oftentimes meet with disappointment.

Some other than an aqueous solution of the chloride, or perhaps some other salt of the alkoloid—as for instance the *oleate* has already gained some reputation—may be discovered to answer all the requirements of the dental surgeon. Experiments will be made in this direction and even though they fail, we will still have the original preparation, which I believe, if patiently manipulated, can prove as great a blessing to us, as it has already to the oculist.

Perhaps I ought to conclude this short paper at once, but feeling as I do that cocaine will hold a brilliant and permanent position in materia medica, I am constrained to respectfully suggest that we should cease calling the compound with hydrochloric acid, a hydrochlor*ate*. The credit that I expected to receive by suggesting the proper name, has, however, been already appropriated by several writers in several scientific journals, received a few days ago.

The last edition of the U. S. Pharmacopœia, unfortunately, when referring to salts of hydrochloric acid, drops the time honored though meaningless "muriate," and adopts in its stead, the old, disused, and misleading "hydrochlor*ate*."

Although Professor Atfield uses in this connection, the word hydrochlorate, his definition of the manner in which salts are named, contradicts his own rules of nomenclature. For instance, he says, "a salt whose name ends in *ate* contains a *compound* acidulous radical, whose name ends in "ic"; and again "a salt whose name ends in *ide*, contains an *element* for its acidulous radical." And "the acidulous radical of hydrochloric acid (H Cl) is chlorine (Cl)" ergo the names of all salts of hydrochloric acid should end in *ide*. On the other hand, the salts of chloric acid ($H\,Cl\,O_3$) properly terminate in *ate*, inasmuch as their acidulous—or electro negative—radical ($Cl\,O_3$) is not a single element, but composed of two elements.

If the prefix "hydro" must be retained because of the salt being an "addition" product,—or of direct union between the alkaloid and the acid ($C_{17}\,H_{21}\,N\,O_4$, H Cl) without substitution of hydrogen, as in the action of a metal on an acid, the same rule should apply to the salts of alkaloids with other mineral acids; we would thus have morphine *hydro*sulphate, ammonia hydronitrate, etc.

It is obvious therefore, that the prefix "hydro" is not necessary to distinguish the "addition" salts of hydrochloric acid, any more than for the corresponding salts of the other mineral acids, and in order to have uniformity in this regard, the former should be termed "chlorides."

The *Popular Science News* uses the name cocaine *chloride* without any explanation therefor, merely as a matter of course; and the *Drug News* also consistently leans that way, but proposes *hydrochloride* as a convenient compromise between the two extremes. At any rate, if the termination *ate* be misapplied thus, which it is, according to all rules of chemical terminology, we undoubtedly should avoid it; and besides, some of us may discover extraordinary virtues resulting by combining cocaine, or some other alkaloid, with chloric acid, forming a compound whose right to be entitled a chlor*ate*, or hydrochlor*ate*, cannot be questioned, and if the name be already improperly given to another, our proposed new remedy must suffer the disadvantages said to accompany those who do not know their own names."

*Dr. Will Taft*—I got some Muriate of Cocaine at the first and have been using it ever since. For obtunding the sensibility of dentine it is of but little use, though upon soft tissues it produces local anaesthesia; it is very good to prevent the nausea often caused by taking impressions of the mouth.

*Dr. Wright*—Dr. Harlan, can Canabis Indica be classed with Cocaine? It has been in use in England of late.

*Dr. Harlan*—I cannot say that Canabis Indica could be classed with preventives of tissue waste. Cocaine belongs to that class, which also includes tea and coffee. Canabis Indica is a powerful obtunder of pain. Some gentleman, with commercial instinct, has tried to patent a process for the painless extraction of teeth by the use of a preparation of this drug; I have no use for such persons as they drag down rather than elevate the standard of dentistry and I want to see it high and shall do all I can to secure that object. This man uses a weak aqueous solution hot, dips his forceps in it and all that sort of thing and then extracts as rapidly as possible; that's all there's in it I imagine. My associate Dr. W. applied the rubber dam to a tooth and bathed the pulp with the tincture I mentioned, and at the end of five minutes I was able to extirpate the pulp without pain. I have used it in several other cases; in one, the rubber was adjusted on six anterior teeth. I applied the canabis indica to two cavities in the lateral incisors and allowed it to remain while I excavated other cavities in the centrals. I then excavated those in the laterals and asked the patient if any pain was felt, the reply was that it was very much diminished; the imagination may have had something to do with it. If we could get the active principle, Canabin, I think we would no doubt get the same results we do from cocaine; I have also experimented with caffeine, theine, and theo-bromine. The alkaloid cocaine is only soluble in 700 parts of water; its salts are very soluble. Merck says that citrate of cocaine made into a paste gives most excellent results. In one case I made a rope of cotton, saturated it with a 4 per cent. solution of cocaine, tied it around the neck of a tooth where I allowed it to remain for ten minutes at the end of which time the tooth was extracted without pain. Dr. Nash injected a

4 per cent. solution near the mental foramen and produced complete paralysis of that side.

*Dr. Sage*—What precautions do you take to prevent the fluids of the mouth from diluting the solutions?

*Dr. Harlan*—Simply a napkin held in the mouth to take up the saliva. In cases where it is necessary to remove necrosed bone at the gingival margin, the gum may be painted with a 4 per cent. solution and the bone successfully removed without causing pain.

A solution of cocaine in eugenol cannot be applied to the gums, as the latter causes too much pain.

*Dr. Will Taft*—Dr. Wright bought up all the cocaine in the market and I would like to know what he did with it all?

*Dr. Wright*—I gave it to my friends. (Laughter.)

*Dr. McKellops*—The great interest I have taken in the reading of Dr. Harlan's and Dr. Cassidy's papers and the discussions following them, is just the thing I go to the meetings of dental societies for, those who stay at home receive no benefit and can not imagine what they have lost. I have used cocaine prepared in this way: $1\frac{1}{2}$ grains of cocaine dissolved in $\frac{1}{2}$ʒ each of chloroform and alcohol, the result is much better and more satisfactory than that of the aqueous solution. I have also tried the fluid extract of canabis indica in a case requiring extraction; I saturated cotton with it and applied it upon the gums surrounding the affected tooth, where it remained for five or ten minutes at the end of which time the extraction was performed with comparatively little pain to the patient.

(Adjourned till afternoon.)

(*To be continued in May No.*)

